# Commercial Opportunities and Ethical Pitfalls in Personalized Medicine: A Myriad of Reasons to Revisit the Myriad Genetics Saga

**DOI:** 10.2174/1875692111311020003

**Published:** 2013-06

**Authors:** Derek So, Yann Joly

**Affiliations:** Centre of Genomics and Policy, Department of Human Genetics, Faculty of Medicine, McGill University, Montreal, QC, Canada

**Keywords:** BRCA genes, breast and ovarian cancer, commercialization, direct-to-consumer advertising, gene patents, genetic diagnostic tests, myriad genetics, trade secrets.

## Abstract

In 1996, the US-based biotechnology company Myriad Genetics began offering genetic diagnostic tests for mutations in the genes *BRCA1* and *BRCA2*, which are linked to hereditary breast and ovarian cancer. Since that time, Myriad has been a forerunner in the field of personalized medicine through the use of effective commercialization strategies which have been emulated by other commercial biotechnology companies. Myriad’s strategies include patent acquisition and active enforcement, direct-to-consumer advertising, diversification, and trade secrets. These business models have raised substantial ethical controversy and criticism, often related to the company’s focus on market dominance and the potential conflict between private sector profitability and the promotion of public health. However, these strategies have enabled Myriad to survive the economic challenges that have affected the biotechnology sector and to become financially successful in the field of personalized medicine. Our critical assessment of the legal, economic and ethical aspects of Myriad’s practices over this period allows the identification of the company’s more effective business models. It also discusses of the consequences of implementing economically viable models without first carrying out broader reflection on the socio-cultural, ethical and political contexts in which they would apply.

## 1 INTRODUCTION

Myriad Genetics is a biotechnology company based in Salt Lake City which holds patents on a number of cancer-linked human genes, including *BRCA1* and *BRCA2*. Myriad offers genetic diagnostic tests for mutations in these genes, which allow physicians and genetic counsellors to better assess their patients’ risk of developing a hereditary cancer and tailor risk management strategies such as prophylactic therapy to that risk profile. Since this method allows treatment to be chosen based on an individual’s own genetic information, Myriad can be considered a good example of a company involved in the growing field of personalized medicine.

Myriad has come under intense scrutiny due to its choice of business models and role in setting a worldwide precedent for companies involved in genetic diagnostics. Yet despite its involvement in these controversies, Myriad has used its exclusive rights to provide *BRCA* testing to become an uncommon commercial success story in the biotechnology industry. This paper presents a concise look back at the legal, commercial and ethical aspects of four business models that have been part of Myriad’s strategy for its *BRCA *diagnostics: the initial patenting phase to clear the market of competitors, the use of direct-to-consumer advertising to expand the market, diversification to other geographic regions and a combination of multiple genetic diagnostic tests, and the transition to a model based on trade secrecy in order to preserve an advantage in test accuracy after Myriad’s *BRCA *patents expire (see Table **1**). By studying the long-term benefits and drawbacks of these strategies, a great deal can be learned about their implications within the developing field of personalized medicine.

### 1.1 Context

Approximately 1 in 800 people carries a *BRCA1 *(breast cancer 1, early onset) mutation and approximately 1 in 500 people carries a *BRCA2 *(breast cancer 2, early onset) mutation. These mutations account for about 15% of the extra risk seen in first-degree relatives of patients with breast cancer. Studies have found that *BRCA1* mutations lead to a breast cancer risk of 40 to 87% and an ovarian cancer risk of about 16 to 68% by age 70, while *BRCA2* mutations lead to a breast cancer risk of 40 to 84% and an ovarian cancer risk of 11 to 27% by that age [1].

Myriad Genetics was formed in 1991 by a group of scientists from the University of Utah’s Centre for Genetic Epidemiology, who had been studying breast cancer susceptibility in a large database of Mormon family pedigrees. Their research was partly funded by the National Institutes of Health (NIH) and partly by private investors such as Eli Lilly and Company [2]. Prior to November 1996, Myriad performed thousands of free *BRCA1* and *BRCA2* tests in order to draw attention to its services [3]. That month, Myriad began marketing a range of BRCA tests called BRACAnalysis^*®*^ to physicians and clinicians, for the price of about $2,400 USD [2, 4]. Since 2006, Myriad has also offered a supplementary BRACAnalysis^®^ Large Rearrangement Test (BART), which costs approximately $700 USD and is ordered by about 65% of BRACAnalysis^*®*^ purchasers [5, 6]. 

These diagnostics are widely seen as a significant breakthrough in personalized medicine, and many clinicians have expressed a preference for Myriad’s accuracy, price and turnaround time [7, 8]. Myriad wished to use BRACAnalysis^®^ to gain a reputation for high-quality genetic tests so that it could build strong affiliations with health care providers, laboratories, and insurers, and eventually begin selling diagnostics for other genes as well [2].

## 2 PATENT ACQUISITION AND ENFORCEMENT

A patent grants its holder a set of exclusive rights over their invention from the date on which the claim is filed [9]*. *Although its legality has been challenged in many jurisdictions including the United States, the patenting of isolated genes and processes involving these genes (if they meet patentability criteria such as utility, novelty and non-obviousness) remains a established practice in most countries with strong biomedical research infrastructure [8, 10-12].

Myriad filed for its first United States patents on the coding sequences, amino acid sequences and associated primers for *BRCA1 *on August 12, 1994 and for *BRCA2* on April 29, 1996. These patents were granted on December 2, 1997 and November 17, 1998 respectively [13, 14]. Myriad also went on to claim the rights to all methods of detecting cancer predispositions by comparing *BRCA* sequences, as well as related techniques such as gene therapy and the ability to test anticancer drugs on cells and organisms genetically modified to have *BRCA* mutations [15, 16]. Myriad also settled a pair of patent infringement lawsuits with another biotechnology start-up, OncorMed (of Gaithersburg, Maryland), by purchasing the opposing patents on a “consensus sequence” for *BRCA1 *[2]. 

Myriad began threatening to enforce its US patents in 1998, shortly after its broadest claims had been granted by the United States Patent and Trademark Office (USPTO) [2]. All but one competing laboratory chose to stop offering *BRCA* tests in response to Myriad’s cease-and-desist letters; however, some continued to provide diagnostics under the guise of “research testing” for patients who could not afford Myriad’s prices [17]. By 1999, Myriad had shut down eight competing diagnostic services, most of which were situated in universities, although it had to resort to litigation in order to prevent testing from being carried out at the University of Pennsylvania’s Genetic Diagnostic Laboratory [8, 17, 18]. Through these methods, Myriad became the only commercial provider of *BRCA* testing in the United States [19].

The commercial success of Myriad’s patent enforcement strategy in the United States is demonstrated by the fact that other privately-owned companies continued to follow in its footsteps even after this model had become criticized by some researchers, academics and patient groups in other countries. For example, Athena Diagnostics (of Worcester, Massachusetts) notably used aggressive patent enforcement to maintain its monopoly on testing for Alzheimer’s disease predisposition, and PGx Health (of New Haven, Connecticut) has done the same for long-QT syndrome [8, 17, 20].

However, Myriad’s business model met with substantial resistance in other jurisdictions, even those in which the company was granted patents similar in scope to its US ones [2]. Myriad was not issued its *BRCA1* and *BRCA2 *patents outside of the United States until several years later; for example, its principal Canadian patents were granted on October 10, 2000 and April 3, 2001, respectively [21, 22]. Over the spring and summer of 2001, Myriad continued its patent-enforcement strategy by sending cease-and-desist letters to diagnostic labs in Canada, this time stating that all *BRCA1* and *BRCA2* tests had to be done either through Myriad or Myriad’s local licensee MDS [2, 11, 12, 23]. At this time, these tests were priced at $3800 CAD by MDS Laboratories, although they had previously been offered by provincial health care systems at a cost of around $1200 CAD [2]. Partly because of this significant markup over the actual cost of performing the tests, it soon became apparent that aggressive enforcement of patents on diagnostic tests was ill-suited to countries with universal healthcare systems. As a result, the decision to send out cease-and-desist letters was condemned by researchers, clinicians and government agencies alike in many first-world countries [2, 8, 11].

According to Myriad, insurance covers more than 90% of the cost of 90% of the BRACAnalysis^®^ tests it performs in the United States [4]. Myriad also provides free *BRCA* testing to uninsured women who meet certain medical and financial criteria through its financial assistance program [24]. But most diagnostic labs in countries with public health care systems are located in hospitals and funded through hospital budgets, and the single-payer system rarely allows the price charged by Myriad to be passed on to patients or insurers [25]. Many health ministries also became concerned that complying with Myriad’s request would set a precedent that would force them to continue buying expensive diagnostics [2]. The resulting resistance to Myriad’s patent enforcement model from health care administrators and government departments caused Myriad to lose a significant portion of its market outside of the United States [17].

Several additional concerns were raised by clinicians and researchers internationally. Although public health care administrators in 2001 had familiarity with kit-based genetic testing, they had not previously experienced business models in which the patent holder retained control of the testing process [2]. Hence, many clinicians feared that sending tests internationally to Myriad would restrict their ability to choose the most appropriate method of testing for each patient [25]. They also worried that having to send tests away would make it more difficult for labs to stay up to date and train new workers to perform diagnostic testing [2]. The idea of sending samples to a private company in Utah, subject to a different set of regulations, also created legal and ethical concerns about quality control and confidentiality [25].

Myriad also made some opponents in the scientific community due to the somewhat erroneous impression among researchers and advocacy groups that Myriad would enforce its patents against academic researchers; in fact, it permitted all basic research on the *BRCA* genes, engaged in over 100 scientific collaborations, and claims not to have pursued any researchers other than those from the University of Pennsylvania’s Genetic Diagnostic Laboratory, which it believed to be using the test for commercial purposes [7, 17, 26]. Myriad even offered its testing services to researchers funded by the National Institutes of Health for less than half the normal price, netting it no profit [3]. 

Although it was stated in some interviews and a written memorandum of understanding with the National Cancer Institute (NCI) that Myriad had no interest in enforcing patents against non-commercial research, its position was poorly communicated to researchers [2, 26]. Some scientists, clinicians and patient groups even feared that Myriad wished to restrict research at public laboratories in order to prevent the identification of flaws in their BRACAnalysis^*®*^ tests [17]. Furthermore, the definition of “commercial” used by Myriad to make its lone case against the University of Pennsylvania was so broad that it technically included any return of results to patients, even those involved in NCI research protocols. Indeed, some physician-researchers have since complained that even though Myriad allows them to perform research on the *BRCA* genes, this puts them in an ethical bind because they are not allowed to tell women who have contributed to the research about their personal results, even when they may be at high risk [4]. Although Myriad did not take action against any other researchers, a few have stated that they did not report the results of their work in order to avoid alerting Myriad to the infringement in the first place [26]. 

Myriad has generally chosen not to address these criticisms in academic literature. Its lack of transparency and its assertiveness in sending cease-and-desist letters may also have made it difficult to follow up on its initial positions and overcome these impressions [2]. The negative response to Myriad may have been influenced further by lingering hostility from many ethicists, researchers and clinicians who had been ethically opposed to for-profit gene patenting [2]. Many breast cancer advocacy groups also saw Myriad’s business model as detrimental to cancer patients. Patients and advocates might have acted based on their moral positions regarding the ownership of genetic material and the application of intellectual property to it, rather than because of specific issues created by Myriad’s commercial strategies [2, 7, 27].

Whatever the underlying causes of this widespread distrust among stakeholders, the resulting negative portrayal of Myriad in the mass media was likely detrimental to its international public image. In 2007, Caulfield *et al*. searched newspaper articles in Canada, Australia, the UK and the United States for references to Myriad Genetics and its *BRCA *patents. Overall, 78% of the articles were predominantly negative, 16% were neutral, and only 6% were positive. Coverage in the United States was the least negative with respect to gene patenting, largely because many stories from Utah reported Myriad’s perspective on the controversy. However, only 56% of the newspaper articles contained multiple perspectives on the issue, indicating the one-sidedness of the discourse [11]. 

As a result of this public outcry, the biotechnology industry attempted to portray Myriad as an outlier rather than a representative of a commercially successful business model [17]. Surprisingly, little hostility has been directed at the University of Utah, even though some of the patents licensed to Myriad remain jointly assigned to the institution.

Myriad has also used patenting to reinforce its market dominance on other diagnostics. For example, its Colaris^®^ test (which currently makes up 70% of the market for hereditary colorectal and endometrial cancer) is not currently protected by patents. Myriad has announced plans to add the gene *MYH *(MutY homolog (E. coli)) to the test, for which it owns the worldwide composition-of-matter and method-of-use patents. This would allegedly allow it to offer a colon cancer diagnostic that is both more sensitive than the competition and protected by patents until 2022 [6]. However, Myriad has recently revealed further plans to combine its BRACAnalysis^®^, Colaris^®^, Colaris AP^®^, Melaris^®^ and Panexia^®^ tests into a single panel called myRisk Hereditary Cancer beginning in 2015. The new test will initially cover twenty-five genes associated with different types of cancer, including eight currently-proprietary ones BRCA1, BRCA2 and MYH [77].

## 3 DIRECT-TO-CONSUMER ADVERTISEMENT

One method which Myriad has employed to expand its market in the United States (and potentially to regain some positive brand recognition in the eyes of the public) is the use of direct-to-consumer advertising (DTCA) on a large scale [28]. Indeed, Myriad labelled its DTCA strategy a “public awareness campaign” whose goal was “to save lives” [29]. Companies which hold a monopoly due to patents have an additional incentive to advertise because, for the duration of the patent, no competitors can share the benefit of raising public awareness. This may be one of the reasons that Myriad chose to conduct DTCA for *BRCA* testing but not for its other products like colon cancer diagnostics, which are also provided by other companies [26].

While drug advertising in the United States is regulated by the FDA, genetic tests are not subject to the same federal oversight [29, 30]. Although Myriad consulted with physicians prior to launching its campaign, there was no premarket review of its advertisements by the government [2, 29]. Unlike prior cancer-related DTCA campaigns, Myriad chose to target a broad population of women with a family history of breast or ovarian cancer rather than a specific group of patients who had already been diagnosed with cancer themselves [26, 28, 31]. 

**Fig. (1):**
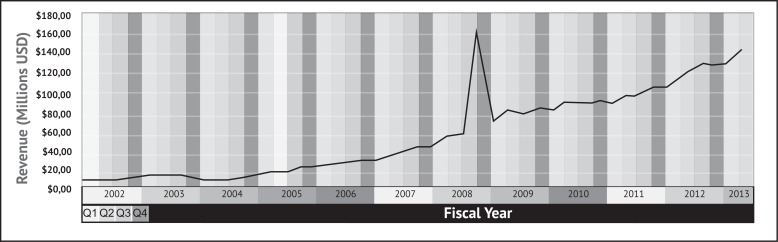
Myriad Genetics’ quarterly earnings from 2002 until the present time. Although generally static in the first half of the 2000s, it has been rising steadily for about eight years. Note the dramatic peak in the final quarter of the 2008 fiscal year (April-June 2008). Data retrieved from the NASDAQ website [74].

Myriad ran its pilot DTCA campaign in Denver and Atlanta from September 2002 to February 2003, targeting both women and physicians through television, radio and print media. One health care provider, Kaiser Permanente Colorado, found that BRACAnalysis^*®*^ referrals increased by over 240% during this period [31]. Myriad’s second DTCA campaign for BRACAnalysis^*®*^ began in Boston, Hartford, Providence and New York in September 2007, and expanded to Florida and Texas that October. Gregory Critchfield, the former president of Myriad’s diagnostic laboratory subsidiary, stated that “the return on investment was sufficiently high that it was worthwhile to go to the next region to extend the benefits of testing to those individuals” [30]. Myriad stock peaked one month after the campaign began, and Myriad’s revenue for the first three quarters of the fiscal year increased by 55% between 2007 and 2008 [2, 29] (see Fig. **1**).

Myriad’s financially-successful efforts also set an attractive precedent for other companies considering DTCA campaigns for genetic tests [29]. The growing attention to personalized medicine has been predicted to increase the prevalence of DTCA for genetic diagnostics, a transition facilitated by liberalized regulations in the United States, modern information delivery platforms, and the participatory decision-making movement [28]. 

Supporters of DTCA claim that it is an effective way of communicating information about health issues to the public and that better-informed patients can take an active role by communicating at an equal level with their health care providers [28]. Awareness of hereditary breast and ovarian cancers can allow women to make use of risk management strategies like MRI screening, early surveillance, anticancer medications such as tamoxifen, or even prophylactic surgery [2, 29, 30, 32-34], as well as giving them the choice to share information with potentially-affected family members [33].

However, Myriad’s campaign also received criticism from clinicians, academics, the mainstream media, and public health agencies such as the Centers for Disease Control [28]. It was frequently claimed that its advertisements emphasized the benefits of *BRCA* testing without mentioning the associated risks. It was also believed by some that Myriad’s DTCA campaign would force insurers to implement more restrictive criteria, limit the amount of attention available to high-risk patients, increase health care costs, and strain genetic counselling systems [28, 29]. Indeed, as a result of Myriad’s broadly targeted DTCA campaign, significantly more low-risk women (whose likelihood of having a *BRCA* mutation was lower than 10%) began to seek testing unnecessarily [35]. According to a survey of physicians, the increase in demand consumed more of their clinic time [28], and it delayed some patients’ access to testing through Kaiser Permanente Colorado [31]. 

Myriad requires that patients receive counselling from their physicians on the benefits and limitations of BRAC Analysis^®^ before a test can be ordered, as well as post-test counselling to determine a management plan [30]. However, although Myriad provides training to physicians it has been found that most lack the familiarity with genetics as well as the time to provide adequate genetic testing, interpretation and counselling [29-31]. Although Myriad produced risk-assessment documents and paid for additional training for physicians on how to perform these steps [2, 29], some experts considered this training still inadequate and expressed concern that it would leave doctors open to a high risk of malpractice litigation. In theory, misinterpretation of results can also result in increased expenditure, unnecessary surgery or false reassurance through the underestimation of actual risk [29, 30].

## 4 DIVERSIFICATION AND TRADE SECRETS

**Fig. (2):**
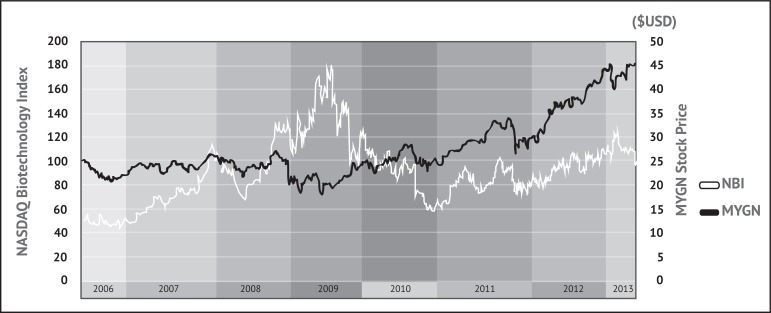
Myriad Genetics’ stock price in comparison to the NASDAQ Biotechnology Index (NBI) since the NBI was begun in 2006. MYGN stock rose steadily from 2006 onward, and unlike most other companies in its field, it continued to rise during the initial phase of the global recession in later 2008 and early 2009. It should be noted that Myriad performed a 2:1 stock split in March 2009. After a period of decreasing price starting around that time, MYGN stock stabilized in late 2010 and began to follow rises and falls in the NBI very closely. Data retrieved from the NASDAQ website [75, 76]. MYGN prices have also likely been affected by Myriad's share repurchase program; since May 2010 the company has bought back $525 million USD of its stock, with an additional $200 million USD authorized for the program in February 2013 [78].

Despite this criticism, Myriad’s tests have continued to sell very well. Myriad stock prices have performed much better than that of most other personalized medicine companies, increasing by 415% from the end of 2003 to the end of 2008 [36]. In fact, MYGN share prices actually increased by 42% in 2008, despite the significant decreases in price seen by every other leading diagnostic company due to the recession [36] (see Fig. **2**). Myriad’s ability not only to survive but to prosper equates to considerable commercial success in the biotechnology industry: although 8,791 start-ups have been spun out of US universities since the passage of the Bayh-Dole Act in 1980, only 3,927 (45%) were still active in the 2011 fiscal year, including 670 created that year [37].

Myriad’s estimated revenue of $595 to $600 million USD for the fiscal year ending June 30, 2013 represents a growth of 20 to 21 percent over last year, and in fact it turned its first profit in October [38, 78]. While Myriad’s diagnostic business had been profitable for years before that, this revenue was previously outweighed by costly and unsuccessful efforts to move into the biopharmaceutical field; its pharmaceutical spinoff Myrexis announced its dissolution in November 2012 [39].

According to Myriad executives, *BRCA* testing has continued to become more valuable as the company increases its penetration into the large, untapped market of eligible customers [40].Yet since a woman only needs to be tested once in her lifetime, this statement is somewhat dubious in the long term given the limited number of regions worldwide in which the population is wealthy enough to afford the current price of testing and in which Myriad remains the sole provider of *BRCA* testing. Indeed, many of Myriad’s business models can be construed as attempts to retain control of a geographically and temporally limited market.

The patents that gave Myriad the greatest advantages over its competitors begin expiring in 2014 and 2015, although executives have stated that the remaining patents out of the original 24 should provide enough protection to ensure exclusivity until at least 2018, and Myriad’s supplemental BART test provides another layer of protection [4, 6, 8, 40]. In preparation for the expiry of these patents, Myriad has begun changing its business model by acquiring new technology and expanding into new markets. 

Although sales of BRACAnalysis^®^ make up about 74% of Myriad’s total revenue and BART testing represents another 11%, the company also offers other profitable cancer predisposition tests, such as the Colaris^®^ test for colorectal and endometrial cancer, Panexia^®^ for pancreatic cancer and Melaris^®^ for melanoma [5, 6, 41]. As previously mentioned, these tests are to be combined into the new myRisk panel along with BRACAnalysis^®^ in 2015 [77]. Myriad has also created new tests such as Prolaris^®^ for prostate cancer aggressiveness and Melapath^®^ for skin biopsy malignancy, as well as thirteen other neurology, dermatology, and autoimmune tests still in development [38]. Myriad has also made approximately 20 collaboration agreements with pharmaceutical partners to develop companion diagnostics for drugs, such as its HRD (homologous recombination deficiency) assay for chemotherapy response, and entered into a partnership with Sanofi to identify diabetes biomarkers [5, 6, 38]. 

In order to increase its market overseas, Myriad has developed a distribution network for its tests in 61 countries [5]. Recently it has been focused especially on the European market, where (partially owing to the controversy over its methods of patent enforcement and a challenge led by the Insitut Curie in 2004 which revoked some of its patent claims in Europe) it does not have complete exclusivity for *BRCA1* and *BRCA2* testing [2, 4, 18, 42]. For example, Myriad built a new diagnostic testing lab in Munich in the spring of 2012 [43]. It has also had some recent commercial success in Australia, where a challenge to the viability of its *BRCA1* patent was dismissed by the Federal Court [44]. An appeal by one of the plaintiffs has already been filed.

An additional challenge which Myriad may have to face within a few years is the rapidly decreasing price of whole-genome sequencing. The price of BRACAnalysis^*®*^ varies by payer, although it is frequently quoted as approximately $3,120 USD, not including the additional cost of a supplementary BART test [4, 7, 8, 26]. However, the cost of a clinical-grade whole genome sequence has recently approached rates of around $4200 USD, and it is expected to continue decreasing in the near future [45, 46]. Hence, this technology could be more of a threat to Myriad’s business model than the loss of its patents, since clinicians would likely prefer to receive information about many genetic conditions for a price comparable to buying a diagnostic test for only one of them [7]. However, interpreting and understanding whole genome data could require significant time, effort, and specialized knowledge on the part of those clinicians. Myriad's newly-announced myRisk panel is projected to cost between $4,000 and $4,500 USD and return results for breast, colon, ovarian, endometrial, pancreatic, skin lung, and prostate cancer risk within 14 days. Since it will also offer a large amount of information for a similar cost and without the effort of interpretation, this panel could prove to be significant competition for whole-genome sequencing in the field of cancer risk diagnostics [77].

The utility of whole genome sequencing as an alternative to Myriad-style diagnostic tests could also depend on how DNA patents are interpreted and enforced [8]. It is not clear at this point whether sequencing an entire genome and using the results to diagnose mutations in the *BRCA* genes would constitute a violation of Myriad’s patents, although a recent paper in *Nature Biotechnology *presents a compelling argument that current forms of whole-genome sequencing do not produce the isolated sequences claimed by most gene patents [40, 46]. A third problem which whole-genome sequencing still faces is the challenge of interpreting thousands of the variants of unknown significance (VUSs) found in each tested individual’s genome [19].

Because the *BRCA1* and *BRCA2* genes both lack mutational hotspots, diagnostic analysis must cover their entire coding sequences, and thousands of different mutations in them have been identified [33]. Although most variants in these genes are either clearly neutral or clearly deleterious, a minority are known as “variants of unknown significance” because there is insufficient epidemiological evidence to interpret them [32-34, 42, 47-49]. Over 1,500 VUSs have been discovered, consisting mainly of missense mutations and splice site mutations [33-34, 42, 47-49]. Approximately 7%-15% of high-risk women tested for a *BRCA* gene are found to have a VUS [32-34, 42, 47-49]. Yet according to a recent study, most do not increase the risk of cancer and “only a very small proportion” of actual deleterious mutations are VUSs [48].

Nevertheless, finding accurate and efficient methods to classify VUSs is becoming an important health issue, since their interpretation is necessary to guide genetic counsellors in identifying risk levels and choosing appropriate management strategies [32, 47, 50, 51]. This analysis typically requires knowledge of VUS frequencies in different populations and ethnicities, knowledge of, detailed pedigrees indicating co-segregation and co-occurrence in family members, knowledge about co-occurrence between VUSs and known pathogenic mutations, tumour immunohistochemistry, and functional analysis when possible [52]. Hence, a large amount of data is required to interpret results from tests like BRACAnalysis^*®*^.

Most patients who use a* BRCA* test voluntarily provide Myriad with their family medical histories in order to aid in risk assessment, and the company obtains even more data through its policy of free testing for the family members of patients with newly-discovered VUSs [19, 52]. By retaining this information, Myriad has built up a large proprietary database of information about VUSs. Although it initially contributed this data to public databases such as the Breast Cancer Information Core mutation database, Myriad stopped making major data deposits in November 2004, and also stopped contributing to the papers that developed the principal public system for VUS evaluation after 2007, fearing that this information could potentially benefit its competitors [2, 19, 40]. Although Myriad described its approach to “calling” VUS results in papers published in *The American Journal of Human Genetics*, *Community Oncology*, *Cancer*, *Cancer Research* and other journals, it did not include its analytic algorithms or sequence data. This choice ran counter to pre-existing recommendations by the National Academies, as well as the *Uniform Requirements for Manuscripts Submitted to Biomedical Journals* which would later be published by the International Committee of Medical Journal Editors [19]. Indeed, another way in which Myriad has adapted to the upcoming expiry of its patents is by instituting a policy of keeping VUS data as trade secrets [19, 40].

Myriad’s policies make it extremely difficult for other geneticists to construct and update the algorithms necessary to accurately interpret the effects of genetic variants [51]. This gives Myriad a competitive advantage over other test providers by allowing it to interpret VUS results with much greater accuracy [19]. Although this information generally allows Myriad only to reclassify VUSs as neutral and not as pathogenic, Myriad has produced market research reports which claim that its tests produce uncertain results only 3% of the time, compared to between 20 and 40% for most European laboratories [19, 40]. Hence, even in jurisdictions where Myriad’s patents have expired or been overturned, it is still able to claim that its tests are better than those of its competitors. Regarding Myriad’s entry into Europe, president and CEO Peter Meldrum told analysts in January 2011 that “I would not want to go into a new market in a heavy-handed fashion, trying to enforce patents”. He stated that Myriad would instead rely on the advantage granted by its faster turnaround and “vastly superior information” [40]. Hence, the advantage in gathering data granted by Myriad’s US patents will likely permit Myriad to extend its dominance over the market long after the patents themselves have expired [19].

The European Society of Human Genetics (ESHG) issued a statement claiming that Myriad’s new strategy would give them an unfair advantage against European academic institutions and hinder the progress of personalized medicine [51]. Some critics have also expressed concerns that the trade secret model would prevent independent verification of tests’ validity, forcing patients to choose between analyses based on less data and more complex ones whose accuracy and completeness have not been critiqued by the broader scientific community [19, 42]. This model has also been criticized because the research which led to Myriad’s patents was partly funded by public dollars but the resulting data has not been adequately shared with the scientific community. Thus, the ESHG called for policymakers to re-evaluate regulations and reimbursement policies to ensure that all clinically-relevant data are made public [51]. The response to Myriad’s entry into Europe may indeed be used to set a precedent for policies on access to mutation data, across all types of genetic analysis [19]. 

Yet Myriad has a number of advantages which will endure even if it are forced to drop its patents or accommodate new data access policies, including its efficient laboratory, pre-existing network of health professionals and payers, and trained sales force [19]. Myriad also has a measure of brand recognition among US customers as evidenced by the effectiveness of its advertising campaigns, and its respected accuracy, turnaround time and customer service should contribute to its continued commercial success [6].

## 5 DISCUSSION

Since its formation, Myriad Genetics has been at the forefront of the movement towards personalized medicine and the resulting controversies associated with the commercialization of genetic diagnostic testing. Many of the strategies first employed by Myriad in the context of its *BRCA* tests have been critically analyzed by experts, adopted by other companies, and since become common practice in the biotechnology industry. As such, Myriad has served as an important indicator in assessing the pros and cons of emerging business models in personalized medicine.

Myriad did not so much invent new commercialization models as demonstrate a remarkable capacity for adapting existing ones to ensure its survival. The company entered the personalized medicine field with a fairly traditional patenting and licensing strategy, albeit with restrictive licensing terms and proactive enforcement practices aimed at protecting its market dominance. As the effectiveness of this strategy was gradually weakened by political controversy, legal challenges and the natural expiration of the *BRCA* patents, Myriad responded by diversifying its products, adopting the DTCA advertising approach and looking at the possibility of combining tests and applying new method-of-use patents to make its previously-unpatentable products proprietary. Myriad also decided to make an important shift towards a business model that has gotten increasing attention from private biotechnology companies in recent years: the keeping of genetic data and diagnostic results as trade secrets. This last strategy could have great appeal for private companies working in personalized medicine, especially in Europe, where robust intellectual property protection for databases is recognized by law [53]. At a time where governments and investors have been increasingly worried about the absence of concrete, marketable clinical applications of the genomic revolution, Myriad adopted business strategies that allowed it to deliver the merchandise to researchers and clinicians. It will be unsurprising if Myriad’s strategies continue to act, at least in part, as a guide for how a commercial company involved in personalized medicine can effectively exploit its technologies for financial benefit. However, a side effect of leading an industry is the potential to serve not only as an example of commercially effective strategies but a warning against unsuccessful ones.

It is worth noting that all three of the principal business models discussed in this paper (patent enforcement, direct-to-consumer advertising and trade secrets) have raised ethical concerns relating to two particular areas: Myriad’s methods of securing dominance over the market, and the conflict between the corporate need for profitability and public health. Whereas the aim of patenting and enforcement is to prevent competing companies from offering commercial *BRCA *diagnostics, direct-to-consumer advertising is meant to build brand fidelity and data secrecy is meant to prevent other companies from practically testing for *BRCA* mutations. All three of these practices can help maintain Myriad’s exclusivity over a prolonged period of time, and some could even be seen as attempts to extend its bundle of exclusive rights beyond the twenty-year period of time designated by the patent system.

Meanwhile, much of the criticism directed towards Myriad can be attributed to concerns (especially in jurisdictions with public health care systems like Canada and Western Europe) that these strategies have enabled the company to set a standard of care that maximizes its revenue but results in health inefficiencies such as cost-ineffectiveness, lack of external quality control, public misinformation, potential overutilization, and patent abuse.

Myriad’s opponents have shown singular persistence in denouncing the company’s business practices in scientific and public media. Critics have sometimes benefited (through publications in key scientific journals, popular media, peer recognition, consultation fees, awards, positive press, etc.) from the high profile taken by the debate and their personal stand against Myriad. Moreover, the intense media coverage led to a polarization of the respective positions. These two factors, as well as the unpopularity of gene patenting, could have contributed to the singling out of Myriad as the representative of biotechnology companies that patent and commercialize human genomic innovations. This, in turn, made it increasingly difficult for Myriad to effectively communicate its position on key ethical and social issues associated with its practices.

The preceding comments should not be read as an attempt to set aside the substantial ethical issues raised by some of Myriad’s practices. Although personalized medicine entrepreneurs would do well to take inspiration from Myriad’s business acumen and adaptability, they must also consider the ethical and social pitfalls the company encountered and the associated costs. It is possible that these difficulties did not lie inherently with the different business models used by Myriad, but were instead created by the heavy handed approach sometimes used to implement and promote them. 

The fact that Myriad unintentionally managed to become the standard example of a biotechnology company embroiled in legal and ethical controversy has undoubtedly led other companies to pursue more cautious patent enforcement strategies while paying more attention to maintaining their public image [54]. It must also be remarked that it is challenging to extrapolate from its commercial success in the diagnostic market of breast and ovarian cancer to other diseases with entirely different patient populations and demographics. The adoption of similar strategies by companies such as Athena Diagnostics and PGx Health can make it tantalizing to draw lessons from Myriad with respect to the entire diagnostics industry. Yet it is key to remain aware of these differences between tests and target populations, as well as the differences between markets with different types of healthcare system, when analyzing these companies' successes and failures.

### 5.1 Myriad Genetics and Personalized Medicine

Given the evident importance of Myriad in setting both positive and negative examples for the biotechnology industry, it is important to examine the extent to which the business models used for BRACAnalysis^*®*^, and the potential ethical concerns associated with them, hold true for other examples of personalized medicine.

“Personalized medicine” is concerned with the customization of patients’ health care to their medical needs, particularly on the basis of their genetic information. In personalized medicine, genetic diagnostics are typically used to determine the viability of genotype-dependent treatments for patients who have already been diagnosed with a certain disease. Hence, this practice extends beyond mere diagnostic testing to the incorporation of personalized therapeutic measures based on the information uncovered this way. Therapeutic products released alongside genetic tests in this way are referred to as “companion drugs” [55, 56].

Myriad’s BRACAnalysis^*®*^ test differs from this norm in two major ways: it is targeted to people at high risk (women who have a family history of breast or ovarian cancer but who are currently healthy) rather than those who have already developed the disease; and it is not marketed alongside a companion drug. Although many women who are found to be at a high risk of breast cancer are prescribed the drug tamoxifen, this drug is not offered by Myriad and the prescription does not depend directly on the genomic information uncovered by BRACAnalysis^*®*^. Many women also choose to undergo prophylactic mastectomies rather than rely on medication, a catchall treatment which falls outside the scope of pharmacogenomics. However, Myriad has recently moved in the direction of the personalized medicine business model by partnering towards the development of a number of diagnostics for companion drugs, such as the HRD test mentioned in the previous section.

Two of the most established drugs used in personalized medicine are trastuzumab (a monoclonal antibody offered as Herceptin^*®*^ by Genentech alongside the HercepTest™ diagnostic by Dako) and imanitib mesylate (offered as Gleevec^*®*^ or Glivec^*®*^ by Novartis). A good comparison can be drawn between the HercepTest™ and BRACAnalysis*^®^,* since both products were developed by start-up companies at approximately the same time for the treatment of the same type of cancer, resulting in a greatly overlapping customer base [57].

The HercepTest™ is an immunohistochemical test used to determine whether a tumour overexpresses human epidermal growth factor receptor 2 (HER2) protein, which occurs in approximately 20% to 30% of breast cancer cases and is associated with poorer prognosis [58, 59]. FDA approval for the combination of the HER2 expression diagnostic and therapeutic antibody was granted in 1998, making it the very first combination pharmacogenomics product, in the same year Myriad began threatening to enforce its patents on *BRCA1* and *BRCA2* [55]. Yet unlike Myriad, Genentech had no competitors to remove before taking advantage of its patents. 

Herceptin^®^ and Gleevec^®^ became very financially successful, both largely as a result of being offered at high prices. According to a recent paper comparing Myriad and Genentech, Myriad was one of the first companies to apply a “blockbuster” financial model to a diagnostic test at a time when the strategy of charging high prices for a patented product was mainly used to recoup the extremely high cost of developing pharmaceuticals [57]. Genentech has also been criticized for the cost of Herceptin^®^: one year of treatment can cost $54,000 USD [60]. As a result, many countries with single-payer health systems (and even some without, such as the United States) have raised doubts over the costs and benefits of funding Herceptin^®^ [61]. Yet unlike the case of BRACAnalysis^®^, Herceptin^*®*^ was readily approved for funding in Canada due largely to patient enthusiasm over its therapeutic benefits [62]. Hence, the coupling of a diagnostic to a potentially life-saving medicine can be very commercially useful to biotechnology companies which offer personalized medicine products.

Yet as previously discussed, since Myriad was only offering a diagnostic test they were able to conduct their business without having to comply with the FDA’s regulations for drug development and advertising. Since the HercepTest™ is targeted to a specific subgroup of cancer patients rather than the general population, there was no real necessity for Genentech to conduct DTCA. However, it did conduct a significant information campaign to promote the HercepTest™ among physicians, which ended up raising many of the same concerns that Myriad encountered in their DTCA program: a 2005 study found that one fifth of European physicians surveyed felt that *HER2* tests were difficult to analyze, despite the availability of additional training from their manufacturer [63]. Apparently, the issue of educating physicians about the use of new diagnostics has been faced by many types of companies involved in personalized medicine.

The patent on Herceptin^*®*^, like Myriad’s earliest patents, expires in the European Union in 2014. Roche has also been focusing on diversification within the field of cancer drugs, including a number of new breast cancer medications [64]. The Denmark-based Dako Corporation, which has been offering the HercepTest™ under license from Genentech, was bought by Agilent in 2012 for $2.2 billion USD [65]. With the expiry of the Herceptin^*®*^ patent approaching, Dako has been successful in having the HercepTest™ approved by the FDA for new companion drugs, including Genentech’s breast cancer medicines Perjeta^*®*^ (pertuzumab) and Kadcycla™ (ado-trastuzumab emtansin) [66, 67]. This strategy highlights the potential flexibility of companion drugs and diagnostics for personalized medicine in the face of patent expiry. 

Novartis’ drug Gleevec^*®*^ was approved by the FDA in 2001 as a treatment for chronic myeloid leukemia (CML). CML is characterized by a Philadelphia chromosome resulting from a reciprocal translocation between chromosomes 9 and 22, containing a chimeric gene called *BCR-ABL*. This gene produces a tyrosine kinase protein seen in 95% of CML patients, which is inhibited by Gleevec^*®*^. Hence, the presence of this translocation is used diagnostically as well as therapeutically to measure patients’ response to the drug. Gleevec^®^ also inhibits another kinase called c-KIT, which has enabled its use for patients who are diagnosed with *KIT*-positive gastrointestinal stromal tumours [55, 56, 68, 69]. The drug has been extremely successful commercially, and dominated the leukemia market in 2011 with sales of $4.7 billion USD [70].

Gleevec^*®*^, like BRACAnalysis^*®*^ and Herceptin*^®^, *has come under fire for being overly expensive. When introduced to South Korea in 2001 for the proposed price of 2,886,000 won per month (roughly $2200 USD and 30 times the tablets’ production cost), activist groups claimed that Novartis was trying to overcharge Koreans. Although it offered to pay a third of the outpatients’ costs through the Novartis Patient Fund, critics claimed that the company had based its pricing on the precedent of wealthier countries and had failed to take into account the fact that Korean patients are required to pay 30% of their total medical costs themselves [71]. Like Myriad, Novartis may have unintentionally stirred up controversy by failing to account for differences in health care systems and socioeconomic factors in their commercialization strategy.

In 2002, civic groups filed a request to waive Novartis’ licence for Gleevec^*®*^ so that South Korean companies would be able to produce the drug for lower prices [71]. Although this motion was not successful, business models that are ill-aligned to the realities of health care in client countries can sometimes lead to the loss of exclusivity in these countries, as Myriad discovered in Canada and the European Union. Also like BRACAnalysis^*®*^ and Herceptin*^®^,* the patents on Gleevec^*®*^ begin to expire in 2014, meaning that Novartis has begun to advocate the use of its other leukemia drugs in order to maintain its dominance in that market [70]. These examples indicate a number of ways in which the business models and ethical issues associated with BRACAnalysis^*®*^ have been mirrored in other personalized medicine products for cancer over a similar time frame. 

Recent trends in the field of personalized medicine such as multiplex testing and drug repurposing have promised to facilitate the increased use of pharmacogenomics in health care. Multiplex testing allows clinicians to easily retrieve information about a large number of potential biomarkers; for example, Roche’s AmpliChip CYP450 Test uses approximately 240 unqiue probes to detect variants in the *CYP2D6* and *CYP2C19* genes, which may affect a patient’s metabolism of tamoxifen when that drug is used to treat breast cancer [72]. Multiplex tests can also be easier patents to defend as machines or methods of use, given that they typically represent a novel piece of technology.

Drug repurposing, which involves the introduction of pre-existing drugs for new clinical applications, is a cost-efficient way to improve the availability of treatments for the many disease subgroups uncovered through the use of genetic diagnostics. This has the potential to be a particularly useful technique in the context of health care, since personalized medicine often allows the use of drugs for patients with specific genotypes that would not be considered sufficiently effective to be approved for the general population [73]. Given the rising importance of personalized medicine, it will be important for biotechnology companies and policymakers alike to consider the successes and failures of the busiess models used for many of its best-selling products over the past fifteen years.

## CONCLUSION

Although the ongoing legal struggle over Myriad’s patents on the *BRCA* genes is an enormous topic in its own right and beyond the scope of this paper, it is also worth mentioning that Myriad is poised to set yet another important precedent for the field of personalized medicine and for intellectual property law when the validity of its gene patents is decided by the Supreme Court of the United States. It is not likely that the results will greatly affect Myriad’s business plan, as those patents will be almost expired by the time they could be affirmed or overturned. Myriad’s choice to move toward a trade-secret based model, build brand recognition through direct-to-consumer advertising, and diversify into new products and jurisdictions may even be seen as preparations for the potential loss of patent-based exclusivity for genetic tests in the United States. In this sense, Myriad has done well in protecting itself from the potential fallout of a controversy it first engendered. It will be interesting to see if public and academic attention moves away from Myriad as BRACAnalysis® is discontinued and its business models continue to evolve with the development of personalized medicine.

In conclusion, a study of the business strategies of Myriad Genetics and their ethical implications provides important information about the evolving context of genetic testing in the biotechnology industry and personalized medicine in particular. By understanding the commercial and ethical successes and failures of this company, we can gain a better understanding of the social and economic forces that shape an increasingly important component of modern health care.

## Figures and Tables

**Table 1.** Overview of Myriad Genetics business models.

	Patent Acquisition and Enforcement	Direct-to-Consumer Advertising	Trade Secrets
**Time frame**	USA from 1998-1999 Internationally c. 2001	Denver and Atlanta in 2002-2003 Northeastern US, Florida and Texas in 2007-2008	2004-present
**Strategy**	Broad patent claims involving genes *BRCA1* and *BRCA2* Purchased opposing patents Sent cease-and-desist letters to labs offering commercial testing Required that tests be done by Myriad or local licensee	Targeted ads to women with family history of breast cancer Described *BRCA* testing as empowering and good for public health Required that physicians offer counselling to patients before and after testing	Accumulated data on variants of unknown significance (VUS) through testing Stopped depositing VUS data in public databases Stopped publishing analysis algorithms
**Ethical concerns**	Increased cost to public health systems could affect patient care Loss of control over testing by public labs and universities Potential to prevent return of results to patients Potential to prevent research on patented genes Requirement to send tests to Utah for analysis created trust/confidentiality issues for stakeholders outside US	No premarket review of ads Potentially misleading ads emphasized benefits over risks Potential to create unnecessary public anxiety Increased attention consumes clinic time Physicians not prepared to analyze tests or provide counselling	Potential extension of exclusivity beyond period granted by IP law Data kept secret despite partial public funding Peers unable to critique whether tests meet clinical criteria
**Commercial impact**	US competitors stopped offering clinical tests Mainly negative portrayal in international media damaged public image Resistance led to loss of most of EU and Canada markets Healthy revenue generated from US consumers	Increase in number of women seeking referral Increased revenue and stock price following campaign	Potential extension of exclusivity beyond period granted by IP law Secret information may be used as a basis to claim higher test accuracy than competitors

## ABBREVIATIONS

BART: = BRACAnalysis^®^ Large Rearrangement Test
*BCR-ABL*: = *“*breakpoint cluster region” gene fusion with “C-abl oncogene 1, non-receptor tyrosine kinase”
*BRCA1*: = breast cancer 1, early onset
*BRCA2*: = breast cancer 2, early onset
*KIT*: = v-kit Hardy-Zuckerman 4 feline sarcoma viral oncogene homolog
CAD: = Canadian dollars
CIHR: = Canadian Institutes of Health Research
CML: = chronic myeloid leukemia
*CYP2D6*: = cytochrome P450, family 2, subfamily D, polypeptide 6
*CYP2C19*: = cytochrome P450, family 2, subfamily C, polypeptide 19
DTCA: = direct-to-consumer advertising
ESHG: = European Society of Human Genetics
FDA: = Food and Drug Administration
*HER2*: = human epidermal growth factor receptor 2
HRD: = homologous recombination deficiency
MYGN: = Myriad Genetics, Inc. stock symbol
*MYH*: = MutY homolog (E. coli)
NBI: = NASDAQ Biotechnology Index
NCI: = National Cancer Institute
NIH: = National Institutes of Health
USD: = US dollars
USPTO: = United States Patent and Trademark Office
VUS: = variant of unknown significance
